# Evaluation of the analytical performance of a point‐of‐care analyzer for the measurement of feline serum thyroxine concentration in comparison with a chemiluminescence enzyme immunoassay

**DOI:** 10.1111/vcp.13416

**Published:** 2025-02-10

**Authors:** Agnes C. Gläsel, Kristina Weiler, Alexander Pankraz, Natali Bauer

**Affiliations:** ^1^ Department of Veterinary Clinical Sciences, Clinical Pathology and Clinical Pathophysiology Justus‐Liebig‐University Giessen Germany; ^2^ Biocontrol, Bioscientia Healthcare GmbH Ingelheim Germany

**Keywords:** analytical validation, cat, immunofluorescence assay, thyroid gland, thyroxine

## Abstract

**Background:**

Total thyroxine (TT4) measurement is used to assess thyroid status in cats.

**Objectives:**

The aim of the prospective study was to evaluate the analytical performance of the point‐of‐care analyzer (POCA) Immuno AU10V using the v‐T4 test kit for feline TT4 measurement. Additionally, method comparison with a benchtop analyzer (IMMULITE 2000) was done.

**Methods:**

Validation included linearity, inter‐ and intra‐assay precision, precision near the lower limit of quantification (LloQ), and interference testing for hemoglobin, lipid, and bilirubin. Correlation and bias were assessed.

**Results:**

Linearity was given within the dynamic range. Coefficients of variation (CV) were ≤4% near the LloQ as well as for intra‐and inter‐assay precision. No interference was observed for lipid and bilirubin, while hemoglobin caused a negative bias of 28%. Method comparison included 74 samples within three TT4 concentration ranges (0.5–3.7, >3.7–5.13, >5.13–8 μg/dL). Correlation between POCA and reference method was excellent (*r*_s_ = 0.95) with a slight proportional bias of 4.5%. TE_obs_ was between 7.0% and 9.8%. Despite substantial agreement, discordant results on thyroid status occurred in 15% of samples.

**Conclusions:**

The analytical performance of the POCA was excellent, as was its correlation with the reference method. Except for the interferent effect of hemoglobin, the TE_obs_ was <TE_a_ for all analyses. Analysis of severely hemolytic samples is not advised. However, the relatively small dynamic range of the POCA precludes quantitative analysis of samples with TT4 >8 μg/dL, and de novo reference intervals need to be established.

## 1 INTRODUCTION

Hyperthyroidism is the most frequent endocrinopathy of the adult and geriatric cat.^1^ Measurement of total tetraiodothyronine/thyroxine (TT4) concentration is frequently performed to evaluate thyroid function.^2^ The TT4 results are used to screen for hyperthyroidism and therapy monitoring.

A commonly used analytical method for TT4 measurement is a chemiluminescence enzyme immunoassay (CLEIA) run on benchtop analyzers. Due to high acquisition and maintenance costs, the CLEIA is restricted to large laboratories. Demand for fast, cost‐effective, and reliable TT4 measurement has led to the development of assays suitable for point‐of‐care analyzers (POCAs).^3^, ^4^, ^5^, ^6^, ^7^

To date, few studies have evaluated the analytical performance of feline TT4 measurement with a POCA.^4^, ^5^, ^6^ The assay methodologies of the evaluated tests include two ELISA Snap tests, a dry‐slide ELISA system, a fluorescent enzyme immunoassay (FEIA), and an automated immunoassay.^3^, ^4^, ^5^, ^6^, ^7^, ^8^ The extent of the evaluation in these previous studies varied from method comparison only to more extensive investigations that included evaluation of precision and rarely also diagnostic agreement of thyroid status. None of these studies included interference testing.

Most of the evaluated assays showed acceptable to good analytical performance and good correlation with respective reference methods.^4^, ^5^, ^6^, ^7^ Two studies included a comparison of diagnostic agreement concerning thyroid status (hypo vs normo vs hyperthyroid). One study using a dry‐slide ELISA found very high agreement (>96.8%), while a first‐generation snap ELISA incorrectly classified 50%–65% of feline samples compared with a validated radioimmunoassay (RIA).^3^, ^4^ The authors of the second study do not clearly state whether this discordance was due to false‐positive or false‐negative results. Possible causes for discordant results of the snap ELISA might have been a high imprecision (28%) and the presence of a proportional bias, which led to an overestimation and underestimation of samples with a low and high TT4 concentration, respectively.

With the Immuno AU10V analyzer (Fujifilm Cooperation, Tokyo, Japan), an additional POCA, able to perform a feline TT4 assay (DRI‐CHEM, Immuno AU10V v‐T4 test, Fujifilm Cooperation, Tokyo, Japan), has become available.^9^ In addition to feline and canine TT4, the Immuno AU10V can measure feline and canine bile acids, canine cortisol, canine thyroid‐stimulating hormone, and progesterone, as well as feline serum amyloid A.^9^, ^10^, ^11^

The principle of the Immuno AU10V assay is a competitive immunofluorescence assay using surface plasmon‐enhanced fluorescence (SPF) and surface plasmon resonance.^12^

Briefly, the assay principle is as follows: a thin gold film is irradiated at a specific angle to create evanescent waves within the metal. These evanescent waves resonate with compressional waves of free electrons of the gold film, so‐called surface plasmons. This leads to an enhancement of the photoelectric field near the surface of the gold film (near‐field light). When fluorescent particles enter the near‐field light, their fluorescent signal is amplified. Particles outside the near‐field light will not be excited, which results in noise reduction while amplifying the fluorescence signal, leading to a highly sensitive measurement of analytes even at low concentrations.^12^ High sensitivity at low measurand concentrations is essential for analytes like TT4 in hypothyroid dogs.

For the measurement of TT4, soluble fluorescent particles labeled with anti‐T4 antibodies are mixed with the sample. The total T4 present in the sample is bound by anti‐T4 antibodies on the particles. Only the remaining antigen binding sites of the fluorescent particles can bind to T4 immobilized on top of the gold film by bovine serum albumin situated within the near field. This results in their excitation and the emission of fluorescent light which is detected by the Immuno AU10V.^9^, ^12^ As only particles near the SPF are excited, unbound fluorescent particles do not interfere with the measurement, making a washing step unnecessary. Thus, all reaction steps are accommodated within one cartridge, which is suitable for use as a POCA.^9^

The Immuno AU10V has previously been validated for the measurement of feline and canine bile acids.^11^ To the authors' knowledge, the Immuno AU10Vs analytical performance for feline TT4 measurement has not yet been evaluated independently.

Therefore, the aim of this investigation was to perform a method validation study using the Immuno AU10V analyzer (hereafter referred to as POCA). Additionally, a method comparison study between the feline TT4 assay (hereafter referred to as Immuno AU10V) on the POCA and the canine Total T4 assay (Siemens Healthcare GmbH, Erlangen, Germany, hereafter referred to as CLEIA) run on the IMMULITE® 2000 (Siemens Healthcare GmbH, Erlangen, Germany) was performed. Use of the canine TT4 assay on the CLEIA was previously validated for feline TT4 measurement and is frequently used.^5^, ^13^, ^14^, ^15^, ^16^ The CLEIA served as the reference method. The POCAs methodology was shown to provide highly precise and accurate results for other analytes (e.g., feline and canine bile acids).^11^ Additionally, high agreement between POCA and CLEIA for the measurement of canine TT4 was shown by the manufacturer during the assay development process in canine samples.^9^ Therefore, our hypothesis was that the POCA would show high precision for feline TT4 measurement as well as good agreement with the reference method.

## 2 MATERIALS AND METHODS

This prospective study was performed between January 2020 and September 2021.

The assessment included the evaluation of linearity, intra‐ and inter‐assay precision, precision near the lower limit of quantification (LloQ), interferences, and method comparison. Throughout the study, Microsoft Excel (software version 16.72) was used to tabulate data and calculate descriptive statistics.

### 2.1 TT4 measurement with the POCA

Analyses were performed according to the manufacturer's recommendations. Briefly, individually sealed cartridges were allowed to warm to room temperature (approx. 20–23°C), opened, and carefully inserted into the analyzer. The serum was transferred into the provided test tubes. After starting the analysis, calibrations and biochemical reactions were run automatically within each cartridge. Results were available within 10–15 min. If TT4 concentrations exceeded the POCA's dynamic range, they were displayed as >8 μg/dL by the analyzer. A minimum sample volume of 0.1 mL is needed for each measurement.

### 2.2 Method validation

#### 2.2.1 Linearity

To avoid matrix effects caused by excessive dilution with H_2_O or saline, serial dilution was achieved by mixing one pool of serum with a low TT4 concentration (1.1 μg/dL) with another pool containing a high TT4 concentration (7.4 μg/dL). Concentrations of the high and the low serum pools were determined with the POCA. The five‐part dilution recommended by the ASVCP guidelines was expanded to include seven dilution steps in order to generate more datapoints.^17^ Pools were mixed at ratios of 1:0, 4:1, 3:2, 1:1, 2:3, 1:4, and 0:1. The expected concentrations were calculated from the TT4 value measured in the low and high serum pool. According to ASVCP guidelines, the serially diluted samples were analyzed in triplicate with the POCA.^17^ The mean of triplicate measurements and bias were calculated.^17^ Additionally, one aliquot of each dilution was analyzed with the CLEIA (single measurement) to generate a plausibility control. The bias (%) between measured and expected concentration was calculated as follows:

$$\%\text{bias}=\frac{{\text{mean}}_{\text{measured concentration}}-\text{expected concentration}}{\text{expected concentration}}*100$$

The linearity was evaluated with the Deming regression analysis which correlates the observed TT4 concentration against the expected TT4 concentration. This analysis was performed using GraphPad Prism 6 (GraphPad Software, Inc. La Jolla, USA).

#### 2.2.2 Intra‐ and inter‐assay precision

Intra‐ and inter‐assay precision of the POCA was determined by performing replicate measurements and calculating the coefficient of variation (CV). Imprecision was calculated as follows:

$$\mathrm{CV}\%=\frac{\mathrm{SD}}{\text{Mean}}*100$$

Two serum pools, each at three different TT4 concentration levels (low, medium, and high, determined using the POCA), were used. This resulted in six pools overall.

The intra‐assay precision was assessed for the POCA as well as the CLEIA. The six serum pools were divided into two aliquots per pool. Ten replicate measurements of each aliquot were performed within one run with the CLEIA and the POCA, respectively.

For the evaluation of inter‐assay precision of the POCA, pools were divided into seven aliquots per pool. The aliquots were stored at −20°C, and one aliquot per pool was thawed and analyzed (single measurement) each day for seven consecutive days. Sample stability over 35 days of storage at 2–8°C and −20°C has been confirmed previously.^18^

#### 2.2.3 Precision near the lower limit of quantification

Precision near the LloQ was assessed using two serum pools with low TT4 concentrations (1.2 and 2.1 μg/dL, measured by the POCA), which were analyzed 20 times within a single run.

Quality goals derived from biological variation were used to assess precision.^19^ These are calculated from the intraindividual CV (CV_I_), that is, the inherent biological variation of an analyte within an individual. The CV_I_s of TT4 reported previously in healthy cats are 11.4% and 11.6%.^20^, ^21^ This resulted in an optimal CV (CV_opt_), a desirable CV (CV_des_), and a minimal CV (CV_min_) of 2.9%, 5.8%, and 8.7%, respectively which were calculated as follows:

$${\mathrm{CV}}_{\mathrm{opt}}=0.25*{\mathrm{CV}}_{I}$$

$${\mathrm{CV}}_{\mathrm{des}}=0.50*{\mathrm{CV}}_{I}$$

$${\mathrm{CV}}_{\min}=0.75*{\mathrm{CV}}_{I}$$

#### 2.2.4 Interferences

Potential interferences were evaluated by generating three separate serum pools with a TT4 concentration of approximately 3.4 μg/dL. The concentration of each pool was determined with the POCA. Each pool was divided into two aliquots of 480 μL. One aliquot of each pool was spiked with 20 μL of a stock solution containing each of the interfering substances (hemoglobin, lipids, bilirubin), respectively. As no other studies for feline TT4 measurement using in‐house analyzers have so far investigated interfering substances, stock solutions were prepared as described previously for this specific POCA.^11^ The stock solution to simulate lipemia consisted of 10 μL soybean emulsion at a concentration of 100 mg/mL (Intralipid 20%, Fresenius Kabi Canada, Ontario, Canada) and 10 μL double distilled water. This resulted in a final lipid concentration of 4 g/L and corresponds to a mild increase in turbidity.

Stock solution to simulate hemolysis was prepared by diluting 30 mg lyophilized bovine hemoglobin in 0.3 mL 0.9% NaCl, resulting in a concentration of 100 g/L hemoglobin. The final hemoglobin concentration after addition to the sample was 4 g/L. This corresponds to a markedly hemolytic sample.

The stock solution to simulate hyperbilirubinemia contained bilirubin at a concentration of 20 g/L and was obtained by adding 20 mg bilirubin to 1 mL of 0.1 M NaOH. The expected bilirubin concentration after addition to the sample was 800 mg/L, which corresponds to a marked hyperbilirubinemia.

Each remaining aliquot was spiked with 20 μL of the respective diluent used to prepare the stock solution of the interferent. Aliquots spiked with diluent served as controls.

Triplicate measurements were performed in random order. Means of triplicate measurements were calculated. Mean %‐bias between the test and control sample represented the observed interference effect (*d*_obs_) and was determined as

$$d_{\mathrm{obs}}\%=\frac{{\text{mean}}_{\text{test}}-{\text{mean}}_{\text{control}}}{{\text{mean}}_{\text{control}}}*100$$

To fulfill the quality requirements, the *d*_obs_ between control sample and spiked sample had to be below the TE_a_.^20^, ^21^

### 2.3 Method comparison

Seventy‐four serum samples were included in the method comparison study, with approximately equal numbers of samples within the low (0.5–3.7 μg/dL ≙ 6.4–47.6 nmol/L; *n* = 30), medium (>3.7–5.13 μg/dL ≙ 47.6–66.0 nmol/L; *n* = 21), and high (>5.13–8 μg/dL ≙ 66.0–103.0 nmol/L; *n* = 23) TT4 concentration range. The TT4 results generated by the CLEIA were used to assign individual samples to the respective group. The low concentration range in the current study was set from the assay's lower limit of the dynamic range (0.5 μg/dL) to the upper limit of the reference interval (RI) of 3.7 μg/dL provided by the manufacturer. Total T4 results above the RI were divided into mildly (<5.13 μg/dL) increased and moderately to severely (>5.13 μg/dL) increased, as proposed previously.^2^ The upper limit of 8 μg/dL represents the upper limit of the assay's dynamic range.

Residual serum from blood samples obtained from cats presented to the University's clinic, that is, healthy cats for routine check‐up, cats with non‐thyroidal illness, and hyperthyroid cats prior to and 2–6 days after radioiodine therapy.

Samples obtained after radioiodine therapy had to be stored at −80°C for 8 weeks before analysis to ensure that samples did not possess any residual radiation activity.

The study was ethically approved by the University's animal welfare officer and the responsible authority (reference number: V 54—19 c 20 15 h 02 GI 18/17 kTV 6/2019).

The TT4 results obtained with the POCA were compared with those from the CLEIA, which served as the reference method.

Briefly, the CLEIA uses a competitive, solid‐phase, chemiluminescent immunoassay to measure TT4. Patient serum and a reagent containing alkaline phosphatase‐conjugated to T4 are added to a disposable reagent tube. This tube contains a bead coated with murine monoclonal anti‐T4 antibody. After an incubation period, unbound T4 is removed via washing and centrifugation. The chemiluminescent substrate is added and allowed to incubate. Subsequently, chemiluminescence intensity is quantified via a photomultiplier.

All patient samples were divided into two aliquots. One aliquot was sent to an external laboratory (Biocontrol, Bioscientia Healthcare GmbH, Ingelheim, Germany) for TT4 measurement with the CLEIA. During transport to the external laboratory, samples were kept refrigerated. In the meantime, the second aliquot was kept at 4–8°C to replicate conditions during transport. As soon as results from the CLEIA were available, the corresponding aliquot was analyzed with the POCA. Prior to analysis, both aliquots at the respective laboratories were brought to room temperature.

In addition to this protocol, both aliquots from cats taken after radioiodine therapy were stored together at – 80°C for 8 weeks for the sake of radiation safety. After that, they were thawed and analyzed as described above.

Only an insufficient number (*n* = 8) of samples in the medium TT4 concentration range could be collected during the study period. In order to provide sufficient data points for the method comparison in this TT4 concentration range, an additional 13 samples with varying TT4 concentrations within the required range were generated by pooling stored serum samples.

Internal two‐level quality control (low normal, high normal) was performed on the CLEIA before each run of measurements using commercially available control material (Bio‐Rad Laboratories GmbH, München, Germany; Lyphocheck Immunoassay Plus Control).

Correlation and bias between the two assays were evaluated with Spearman's rank correlation coefficient, Bland–Altman plots, and Passing–Bablok regression using MedCalc (software version 16.2.1; Ostend, Belgium). The correlation was considered “very high” for Spearman's rho (*r*_s_) = 0.90–1.0, “high” for *r*_s_ = 0.70–0.89, “moderate” for *r*_s_ = 0.50–0.69, “low” for *r*_s_ = 0.3–0.49, “little if any” for *r*_s_ <0.29, respectively.^22^

For objective interpretation of results, the total observed error (TE_obs_) was calculated and compared with the total allowable error (TE_a_). The TE_a_ of 15% and 14%, respectively, is based on biological variation of the TT4 concentration in healthy cats reported in the literature.^20^, ^21^ Quality requirements were fulfilled when TE_obs_ was below TE_a_. TE_obs_ was calculated as TE_obs_ = % bias + 2 * CV.

Agreement between the two assays for the classification into hypothyroid, euthyroid, and hyperthyroid was compared using Cohens Kappa using the GraphPad QuickCalcs online calculator.^23^ The TT4 results were classified according to the assays´ respective RIs. The RI for the Immuno AU10V (0.9–3.7 μg/dL) was given by the manufacturer and is equal to that published by Peterson et al. (2001) using an RIA.^2^ The CLEIAs RI (1.0–4.0 μg/dL) was generated by the reference laboratory.

Cohen's Kappa and weighted Kappa values were interpreted as follows: <0 = no, 0–0.2 = slight, 0.21–0.4 = fair, 0.41–0.6 moderate, 0.61–0.8 = substantial, and >0.81 = almost perfect agreement.^24^

## 3 RESULTS

### 3.1 Method validation

#### 3.1.1 Linearity

**FIGURE 1:**
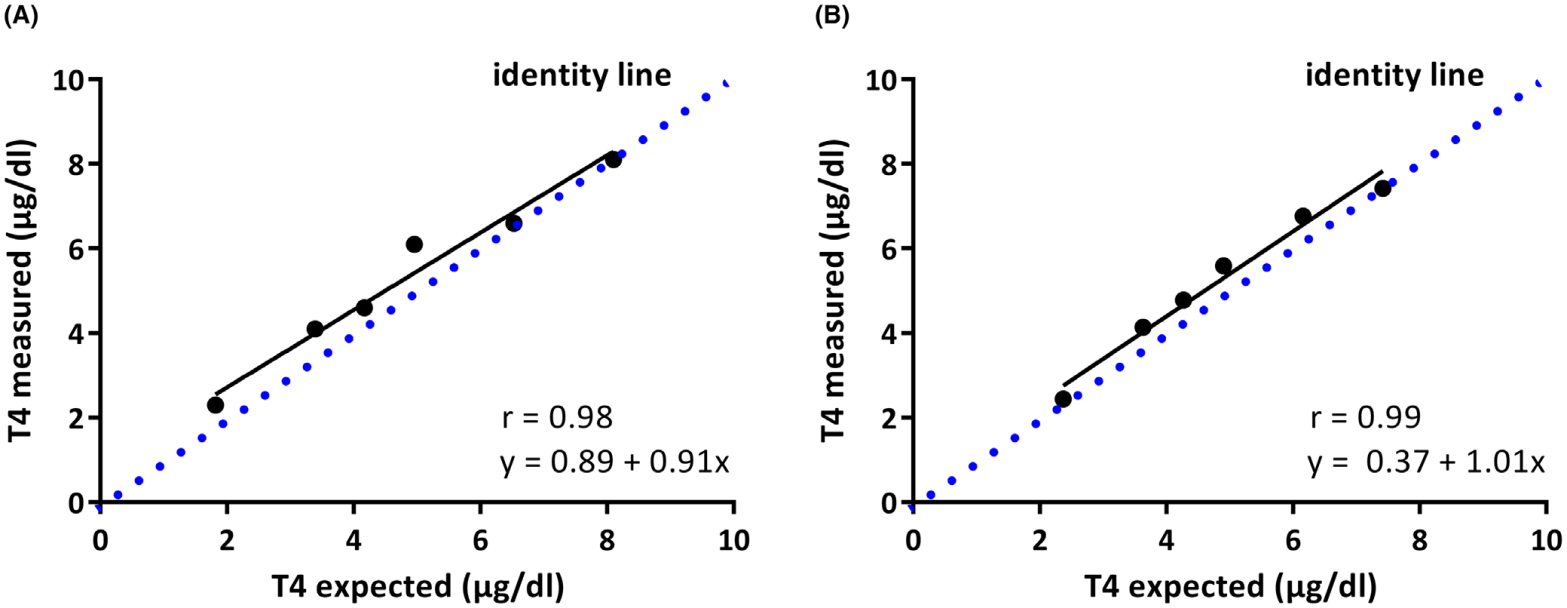
Linearity of total TT4 measurement in feline serum. (A) Linearity obtained by chemiluminescent enzyme immunoassay run on the IMMULITE 2000. (B) Linearity obtained by surface plasmon‐enhanced fluorescence run on the Immuno AU10V.

Linearity was given for both analyzers within the dynamic range of the POCA (Figure 1A,B). Within the dynamic range of the POCA, both CLEIA and POCA showed a very high correlation between expected and measured TT4 concentrations. The bias between measured and expected TT4 concentrations for the POCA was between 3% and 14% and thus TE_a_ (Table 1).^20^, ^21^

**TABLE 1:** Method validation: linearity at seven different levels of TT4 concentrations, achieved by mixing two feline pooled sera (one high, one low TT4 concentration).

Level^a^	Mean measured concentration Immuno AU10V (μg/dL)	Expected concentration (μg/dL)	Measured concentration Immulite^b^ (μg/dL)	Bias %
1	1.11	‐	<0.5	‐
2	2.44	2.4	2.3	3.1
3	4.14	3.6	4.1	14.0
4	4.78	4.3	4.6	12.0
5	5.59	4.9	6.1	14.1
6	6.76	6.2	6.6	9.7
7	7.42	‐	8.1	‐

Abbreviation: TT4, total thyroxine/tetraiodothyronine.

^a^ Level 1: low concentration pool, Level 2: four parts low to one part high, Level 3: three parts low to two parts high, Level 4: one part low to one part high, Level 5: two parts low to three parts high, Level 6: one part low to four parts high, Level 7: high concentration pool.

^b^ Measurements with the IMMULITE 2000 benchtop analyzer at each level were performed as a plausibility control.

#### 3.1.2 Intra‐ and inter‐assay precision

Intra‐assay CVs obtained with the POCA were ≤2.7% and fulfilled the strictest quality goal of CV < CV_opt_ (CV_opt_ = 2.9%) (Table 2).^20^, ^21^ Coefficients of variation for the CLEIA were generally higher, ranging from 2.8% to 7.0%. The CLEIA did fulfill the quality requirement of CV < CV_opt_ only for the highest TT4 concentration (Table 2).

**TABLE 2:** Method validation: intra‐assay precision of feline (*n* = 6) pooled serum TT4 measurements with the Immuno AU10V (point‐of‐care analyzer) and the IMMULITE2000 (benchtop analyzer).

TT4 concentration range (μg/dL)	Sample number	Immuno AU10V (*n* = 10 replicates)			IMMULITE 2000 (*n* = 10 replicates)		
		Mean (μg/dL)	SD (μg/dL)	CV (%)	Mean (μg/dL)	SD (μg/dL)	CV (%)
0.5–3.7	1	2.19	0.06	2.7	2.5	0.13	**5.4**
	2	2.78	0.06	2.0	1.8	0.12	**6.4**
>3.7–5.13	3	3.97	0.05	1.3	4.0	0.26	**6.5**
	4	4.69	0.07	1.4	5.4	0.37	**7.0**
>5.13–8.0	5	5.74	0.15	2.6	6.3	0.30	**4.7**
	6	7.50	0.18	2.4	7.6	0.21	2.8

*Note*: CVs that did not fulfill the quality requirement of CV < CV_opt_ (CV_opt_ = 3% in cats) are printed in bold.^20^, ^21^

Abbreviations: CV, coefficient of variation; CV_opt_, optimal coefficient of variation (0.25 * intraindividual CV); TE_a_, total allowable error; TT4, total thyroxine/tetraiodothyronine.

When applying the less stringent CV_des_ (5.8%), half of the CLEIA's intra‐assay CVs fulfilled this criterion. The minimum quality requirement of CV_min_ (8.7%) was fulfilled by the CLEIA at all concentrations.^20^, ^21^

Inter‐assay CVs of the POCA ranged from 1.5% to 3.6% (Table 3). As such, the CVs for all but one TT4 concentration were below CV_opt_, and all were below CV_des_.

**TABLE 3:** Method validation: inter‐assay precision of feline (*n* = 6) pooled serum TT4 measurements with the Immuno AU10V point‐of‐care analyzer.

TT4 concentration range (μg/dL)	Sample number	Inter‐assay CV (*n* = 7 replicates)		
		Mean (μg/dL)	SD (μg/dL)	CV (%)
0.5–3.7	1	1.54	0.02	1.5
	2	3.00	0.07	2.3
>3.7–5.13	3	3.81	0.09	2.3
	4	4.85	0.10	2.0
>5.13–8.0	5	5.54	0.20	**3.6**
	6	7.15	0.20	2.8

*Note*: CVs that did not fulfill the quality requirement of CV < CV_opt_ (CV_opt_ = 3% in cats) are printed in bold.^20^, ^21^

Abbreviations: CV, coefficient of variation; TT4, total thyroxine/tetraiodothyronine.

#### 3.1.3 Precision near the lower limit of quantification

For the 20‐run intra‐assay precision near the LloQ, the CV of the lowest TT4 concentration exceeded the CV_opt_ but remained below the CV_des_ (Table 4).^20^, ^21^

**TABLE 4:** Method validation: Intra‐assay precision near the lower limit of quantification of TT4 in two feline pooled serum samples with the Immuno AU10V point‐of‐care analyzer.

TT4 concentration (μg/dL)	Sample number	Intra‐assay CV (*n* = 20 replicates)		
		Mean (μg/dL)	SD (μg/dL)	CV (%)
1,21	1	1.21	0.05	**4.3**
2.17	2	2.17	0.05	2.3

*Note*: CVs that did not fulfill the quality requirement of CV < CV_opt_ (CV_opt_ = 3% in cats) are printed bold.^20^, ^21^

Abbreviations: CV, coefficient of variation; TT4, total thyroxine/tetraiodothyronine.

#### 3.1.4 Interferences

No effect was observed for bilirubin at a concentration of 800 mg/L and lipids at a concentration of 4 g/L soybean emulsion. Spiking with 4 g/L hemoglobin resulted in falsely low results for the TT4 measurement. The resulting bias was approximately 28% (Table 5).

**TABLE 5:** Interference testing for bilirubin, hemoglobin, and lipid on TT4 measurement in feline pooled serum samples with the Immuno AU10V.

Interferent concentration	Mean TT4_control_ (μg/dL) ± SD	Mean TT4_test_ (μg/dL) ± SD	Bias (μg/dL)	% Bias	% Bias < TE_a_
Hemoglobin 4 g/L	3.46 ± 0.02	2.50 ± 0.05	0.96	27.8	No
Soybean emulsion 4 g/L	3.47 ± 0.06	3.36 ± 0.00	0.11	3.1	Yes
Bilirubin 800 mg/L	3.40 ± 0.06	3.09 ± 0.03	0.31	9.0	Yes

*Note*: Test specimens (TT4_test_) spiked with the potentially interfering substances were compared to control samples (TT4_control_) spiked with an equal volume of the diluent used for the preparation of the respective stock solution of the interfering substance. Measurements of all specimens were performed in triplicate and random order. Percent bias for the interfering substance was considered acceptable if % bias < total allowable error (TE_a_).

Abbreviation: TT4, total thyroxine/tetraiodothyronine.

### 3.2 Method comparison

Overall, 74 feline samples were included in the method comparison part of the study. These were spread between the three TT4 concentration ranges as follows: *n* = 30 low, *n* = 21 medium, and *n* = 21 high.

**FIGURE 2:**
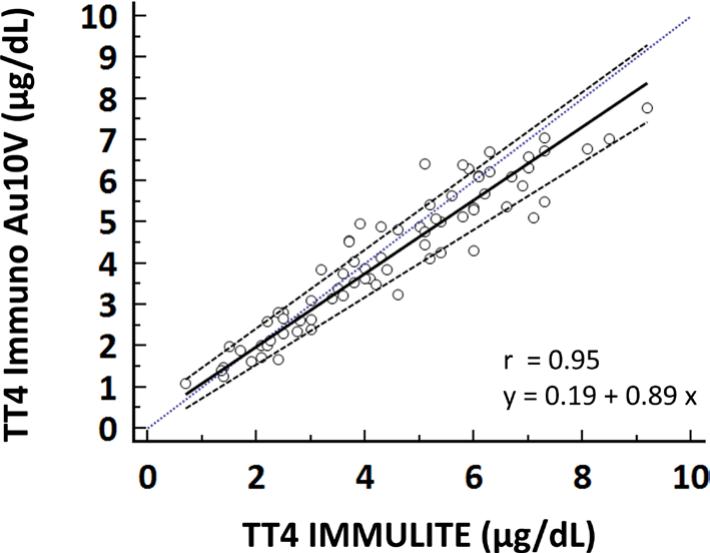
Results of method comparison between the Immuno AU10V and the IMMULITE 2000 (*n* = 74 samples). Passing–Bablok regression line (black line) with 90% confidence interval (dotted line). The blue dotted line represents the identity line.

**FIGURE 3:**
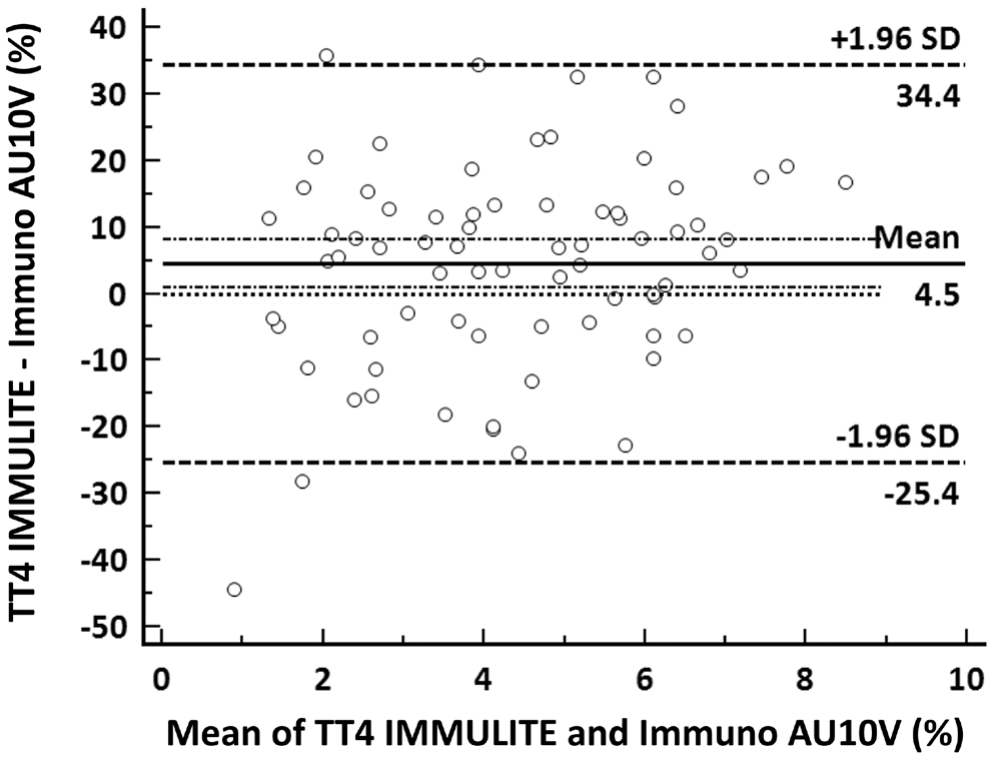
Results of method comparison between the Immuno AU10V and the IMMULITE 2000 (*n* = 74 samples). Bland–Altman difference plot demonstrating mean absolute bias (black line) with 95% of values being encompassed between the thick black dotted lines and its 1.96‐fold standard deviation (thin dotted black lines).

Correlation between the two methods was excellent (*r*_s_ = 0.95). Passing–Bablok regression revealed a small proportional bias with a slope of 0.89 (95% confidence interval: 0.82–0.95) (Figure 2). The intercept was 0.2 (95% confidence interval: −0.09–0.52). A minimal absolute bias of 0.26 μg/dL and a small percentage bias of 4.5% were identified via Bland–Altman analysis. The lower and upper limits encompassing 95% of the observed percentage bias were −25.4% and 34.4% (Figure 3).

The quality requirement of TE_obs_ < TE_a_ was fulfilled at all concentration levels (Table 6).

**TABLE 6:** Total observed error (TE_obs_) for TT4 test run on the Immuno AU10V when compared to chemiluminescent enzyme immunoassay run on the IMMULITE 2000 benchtop analyzer.

TT4 concentration range (μg/dL)	Level	Immuno AU10V vs IMMULITE 2000 (*n* = 10 replicates)		
		Mean bias: 4.5%^a^		
		Mean (μg/dL)	CV (%)	TE_obs_ (%)
0.5–3.7	1	2.19	2.7	9.9
	2	2.78	2.0	4.5
>3.7–5.13	3	3.97	1.3	7.1
	4	4.69	1.4	7.3
>5.13	5	5.74	2.6	9.7
	6	7.51	2.4	9.3

*Note*: TE_obs_ that did not fulfill the quality requirement of TE_obs_ < TE_a_ (14% and 15% in cats) is printed in bold.^20^, ^21^

Abbreviations: CV, coefficient of variation (intra‐assay precision); TE_a_, total allowable error; TT4, total thyroxine/tetraiodothyronine.

^a^ Percentage mean bias calculated from Bland–Altman difference analysis.

Agreement of the classification as hypothyroid, euthyroid, and hyperthyroid, using the CLEIA as the “gold standard,” was substantial (kappa coefficient 0.70, weighted kappa coefficient 0.71) and concurred in 63/74 (85%) of cases (Table 7). “False” low TT4 measurements of the POCA would have resulted in a misclassification of hyperthyroid cats (according to the CLEIA) as euthyroid were present in 3/74 cats (4%). “False” high measurements were observed in 8/74 cats (11%), resulting in classification as euthyroid (1/8) and hyperthyroid (7/8), while they would have been classified as hypothyroid (1/8) and euthyroid (7/8) according to the CLEIA (Table 7).

**TABLE 7:** Comparison of TT4 concentrations and subsequent classification as below, within or above the reference interval (hypothyroid, euthyroid, and hyperthyroid) when using the point‐of‐care analyzer (Immuno AU10V) or benchtop analyzer (IMMULITE 2000).

	IMMULITE 2000		
Immuno AU10V	TT4 below RI (<1 μg/dL)	TT4 within RI (1–4 μg/dL)	TT4 above RI (>4 μg/dL)
TT4 below RI (<0.9 μg/dL)	**0**	0	0
TT4 within RI (0.9–3.7 μg/dL)	1	**26**	3
TT4 above RI (>3.7 μg/dL)	0	7	**37**

*Note*: Number of results in agreement are printed in bold.

Abbreviations: RI, reference interval; TT4, total thyroxine/tetraiodothyronine.

## 4 DISCUSSION

Overall, TT4 measurement on the POCA was easy to perform, and the quality requirements stated in the ASVCP guidelines were fulfilled. Linearity was given within the dynamic range of the analyzer. Intra‐assay precision of the POCA was excellent with CVs < CV_opt_ at all concentrations. Results even surpassed the precision of the reference method (CLEIA).

For the evaluation of precision, generally, a minimum of 20 repeat measurements are recommended. Due to a limited volume of pooled serum, this was not possible in the current study. However, for point‐of‐care analyzers, a minimum of five measurements are deemed acceptable. Most of the TT4 validation studies performed 10 repeat measurements.^25^, ^26^ A higher number of repeat measurements might have led to higher CVs. But as the observed intra‐assay CV at all TT4 concentration levels fulfilled the most stringent quality requirement (CVs < CV_opt_), it seems unlikely that a higher number of repeat measurements would have resulted in unacceptably high CVs.

The intra‐assay CVs reported for previously validated POCAs varied considerably between assays. In previous studies evaluating different concentration levels, CVs ranged from 3.4% to 10% at low TT4 concentrations, thus exceeding the CV_opt_ of 2.9%.^4^, ^7^, ^8^ As expected, reported CVs for medium or high TT4 concentrations were generally lower (2.0%–5.6%).^3^, ^4^, ^7^, ^8^ Except for one assay (first‐generation snap ELISA) with a very high CV of 28% (medium TT4 concentration), most other assays did fulfill the second most stringent quality criterion of CV < CV_des_.^3^

The inter‐assay CV of the Immuno AU10V was lower than most TT4 assays, which ranged from 2.4% to 15%, with the FEIA system achieving the lowest CV at an unknown TT4 concentration.^4^, ^5^, ^6^, ^7^ The higher CVs in the cited studies may, in part, be due to a higher number of repeat measurements (up to 20 repeats over 5 days in one study).

Despite having a CV below that of the CLIA, the CV of one pool near the LloQ exceeded the CV_opt_ while remaining below the CV_des_.^20^, ^21^ However, relatively high CVs are not uncommon at low analyte concentrations, as even a small absolute deviation at low concentrations will result in a higher CV than the same deviation at high concentrations.^27^ This is also reflected in the literature with inter‐assay CVs at low TT4 concentrations ranging from 5.7% to 15% and 4.0% to 8.8% at medium to high TT4 concentrations.^4^, ^7^, ^8^

During interference testing, the addition of 4 g/L of hemoglobin resulted in an unacceptably large bias. Consequently, markedly hemolytic samples should not be analyzed with the POCA. Interference testing was not performed in the majority of validation studies of TT4 measurement with other POCAs.

In a study by Higgs et al, interferences were not systematically evaluated, but the authors noted that mildly to moderately hemolytic or lipemic samples did not cause obvious differences between a fluorescence enzyme immunoassay and the IMMULITE 1000.^18^ Williams and Archer specifically tested for the effect of hemolysis on a human TT4 enzyme immunoassay designed for a benchtop analyzer.^28^ No significant interference of hemoglobin up to a concentration of 8 g/L was detected, but a positive bias of 27% was present at 15 g/L of hemoglobin.

Scarce knowledge is available concerning the impact of interferences on the CLEIA. The manufacturer provides no information on the interference of hemoglobin on the TT4 assay used in this study. But according to manufacturer information for the human TT4 test on the IMMULITE 1000, up to 30 μL/mL of hemolyzed packed red blood cells did not significantly affect the results.

As stated above, the analyzers’ dynamic range for TT4 is between 0.5 μg/dL and 8 μg/dL. During the acquisition of samples for the method comparison part of the study, the majority of samples from hyperthyroid cats was not suitable for the study because TT4 was >8 μg/dL. This observation fits with data from a large population of hyperthyroid cats evaluated by Peterson and colleagues, where approximately half of hyperthyroid cats had TT4 concentrations >8 μg/dL.^2^ In these cats, use of the POCA would have resulted only in a semiquantitative TT4 measurement (TT4 >8 μg/dL). Regardless of knowing the patient's exact TT4 concentration, a diagnosis of hyperthyroidism would still be possible. Nonetheless, in some instances, a quantitative result may be desirable, for example, therapy monitoring or dose finding for radioiodine treatment.

Dilution of the sample is not feasible because the assay principle is affected by the viscosity/matrix of the sample. Thus, the dynamic range may pose a limitation for use of this assay, and TT4 measurement with a different methodology (eg, CLEIA, RIA) may be beneficial in these settings.

When comparing TT4 measurements of the POCA with those of the CLEIA, the correlation was excellent, with only a small proportional bias. Although the mean percent bias, according to Bland–Altman analysis, was small, it has to be noted that the overall range of the percental bias was large.

Substantial agreement was present when classifying patients into hypothyroid, euthyroid, and hyperthyroid based on results from the two analyzers. Nevertheless, discrepancies occurred in 15% of cases. One possible cause for these discrepancies may be the RI used for classifying results from the POCA. The RI provided by the POCA's manufacturer is equal to that established by Peterson et al. using an RIA and might not be ideal for the SPF methodology.^2^ Use of this RI may have led to suboptimal classification of the POCAs results and may have influenced agreement between analyzers. Thus, establishment of de novo RIs would be desirable.^29^ Unfortunately, establishing RIs was beyond the scope of the current study, as the vast majority of cats presented to the clinic during the study period were diseased.

Another cause for discordant classifications may result from the bias detected with the Bland–Altman analysis. Despite the mean bias being low, there was a considerable spread for individual measurements, evidenced by the wide range of values encompassing 95% of the calculated bias (−25.4% to 34.4%).

Despite the fact that great care was taken to ensure equivalent storage conditions for both aliquots used for method comparison between CLEIA (external laboratory) and POCA (in‐house), slight differences in storage time and sample handling were unavoidable (eg, measurement with POCA was performed directly after receipt of CLEIA results). These differences may also have affected the TT4 concentration and, as such, the classification according to thyroid status. However, since TT4 is a stable analyte with little deterioration during storage, it is not expected to have a large effect.^18^

Moreover, the lower precision of the reference method could also have negatively impacted the agreement between analyzers. However, since the CV was below the CV_des_ at most TT4 concentrations, the expected effect was small. Theoretically, the use of a canine assay (as the TT4 assay ran on the CLEIA) might have impacted the agreement between the assays. But, as the thyroxine structure does not differ between species, the use of a canine assay is not expected to affect the analysis, provided that dissociation from the species‐specific binding proteins is effective.^30^

To the authors' knowledge, only two studies have assessed the diagnostic agreement of point‐of‐care assays compared to the respective reference methods, and published results are variable. In a study comparing the classification of a snap ELISA to an RIA, 50%–65% of cases were misclassified with the ELISA.^3^ Another study evaluating a dry‐slide ELISA with an automated enzyme immunoassay showed good analytical performance as well as high agreement between methods.^4^

A limitation of the method comparison part of the current study is the low number of samples with TT4 concentrations in the low normal and hypothyroid range. Although spontaneous hypothyroidism is a very rare condition in cats, iatrogenic hypothyroidism due to medical or radioiodine treatment is not an uncommon occurrence. After radioiodine treatment transient or permanent hypothyroidism is reported in 2%–40% of cases.^31^, ^32^ Thus, confirmation of accurate measurement near the LloQ with enough samples would be desirable.

## 5 CONCLUSIONS

Overall, the measurement of feline TT4 with the POCA was precise and accurate. In addition, the analyzer was easy to use, and results were available within a short turnaround time. However, markedly hemolytic samples will result in falsely low values and should not be analyzed. For the diagnostic interpretation of TT4 results, analyzer‐ and assay‐specific RIs should be established to facilitate optimal classification of results. Additionally, if a quantitative TT4 measurement is needed for a sample that is expected to exceed the dynamic range, a different methodology (e.g., CLEIA) should be employed.

## FUNDING INFORMATION

The author(s) disclosed receipt of the following financial support for the research, authorship, and/or publication of this article. Financial support by Fujifilm Europe was provided to the Justus‐Liebig‐University Giessen. The Dri‐Chem Immuno AU10V and the corresponding cartridges for measurement of T4 (Fuji Dri‐Chem Immuno AU cartridge v‐T4) as well as supplementary material (tubes and tips) were provided by Fujifilm Europe. The company was not involved in planning of the study, analysis, interpretation, or publication of the results.

## CONFLICT OF INTEREST STATEMENT

The authors were funded by Fujifilm Europe; however, the company was not involved in the interpretation or publication of the results.
